# Patient Throughput Initiatives in Ambulatory Care Organizations during the COVID-19 Pandemic: A Systematic Review

**DOI:** 10.3390/healthcare9111474

**Published:** 2021-10-30

**Authors:** Cristian Lieneck, Zo Ramamonjiarivelo, Jennifer Cox, Jack Dominguez, Kendal Gersbach, Edward Heredia, Afroza Khan

**Affiliations:** 1 School of Health Administration, Texas State University, San Marcos, TX 78666, USA; zhr3@txstate.edu; 2 St. David’s School of Nursing, Texas State University, Round Rock, TX 78665, USA; jlc445@txstate.edu (J.C.); jed171@txstate.edu (J.D.); keg162@txstate.edu (K.G.); erh100@txstate.edu (E.H.); aak86@txstate.edu (A.K.)

**Keywords:** ambulatory care, outpatient care, patient throughput, COVID-19, pandemic

## Abstract

Background and objectives: Ambulatory (outpatient) health care organizations continue to respond to the COVID-19 global pandemic using an array of initiatives to provide a continuity of care for both COVID-19 and non-COVID-19 patients. The purpose of this study is to systematically identify the facilitators and barriers experienced by outpatient health care organizations in an effort to maintain effective and efficient patient throughput during the pandemic. Materials and methods: This study systematically reviewed articles focused on initiatives taken by ambulatory care organizations to maintain optimal outpatient throughput levels while balancing pandemic precautions, published during 2020. Results: Among the 30 articles that met the inclusion criteria, three initiatives healthcare organizations have taken to maintain throughput were identified: the use (and enhanced use) of telehealth, protocol development, and health care provider training. The research team also identified three barriers to patient throughput: lack of telehealth, lack of resources, and overall lack of knowledge. Conclusions: To maintain patient throughput during the COVID-19 pandemic, healthcare organizations need to develop strategies such as the use of virtual consultation and follow-up, new guidelines to move patients along the care delivery value-chain, and ongoing training of providers. Additionally, the availability of required technology for telehealth, availability of resources, and adequate knowledge are vital for continuous patient throughput to ensure continuity of care during a pandemic.

## 1. Introduction

### 1.1. Rationale

The novel coronavirus disease of 2019 (COVID-19) is caused by the severe acute respiratory syndrome coronavirus 2 (SARS-Cov-2) and was declared a worldwide pandemic by the World Health Organization in March 2020. COVID-19 has severely affected the world and those with pre-existing conditions, with an estimated 230 million confirmed cases and over 4.7 million deaths [1]. As a result, many countries’ healthcare systems have been overwhelmed while treating COVID-19 patients. Furthermore, the operational work and patient-flow of health care delivery has been disrupted by the unprecedented surge of COVID-19 patients. Health care organizations and their providers often delay the provision of care for non-COVID-19 patients to accommodate COVID-19 patients and to help control the spread of the virus within their respective healthcare facilities. This unexpected and rapid spread of the disease has strained healthcare organizations due to the lack of necessary resources to provide adequate care for COVID-19 and non-COVID-19 patients. Such resources include strains on the availability of health care providers, health care infrastructure issues, and even medication and protective personal equipment (PPE) shortages. These factors have contributed to challenges in processing care for both COVID-19 and non-COVID-19 patients and have negatively impacted patient throughput, defined for purposes of this research study as allowing, “…for the efficient flow of patients through the hospital, ensuring timely and appropriate level of care” [2].

Beyond the simple flow of patients through a health care facility, the research team decided to investigate variables affecting the flow of patients (and/or lack thereof) with an additional, broader purpose—to assess and identify potential best practices or inhibitors of care delivery in the ambulatory care setting as compared to a non-pandemic environment of care. This initiative, while focused on throughput, was evaluated by the research team in an attempt to address questions regarding an alignment of practices and procedures to assist outpatient health care organizations work to sustain somewhat ‘normal’ patient continuity of care operations during the pandemic and required public health protocols. These intended observations were analyzed with regard to several facilitator and barrier constructs identified, including organizational reporting of:

- Frequency of patients seen
- Type of patients seen (example: routine vs. acute, primary care vs. specialty)
- Best practices to provide continuity of care during the pandemic and public health initiatives that potentially restrict normal (non-pandemic) clinic operations

The research team focused on these throughput initiatives at this broad level in order to be as inclusive as possible due to the limited research surrounding patient throughput in the ambulatory care setting during the COVID-19 pandemic. Identified facilitators and barriers to outpatient throughput initiatives can further assist ambulatory care clinics in their ongoing challenges to continue patient care in a more efficient manner.

Several systematic reviews on patient throughput/patient flow have been conducted. Some of these reviews looked at the relationships between lean health care and patient flow [3], the impact of scribes on patient throughput [4,5], strategies used to improve patient flow [6,7], the role of computer simulation modeling on patient flow [8], and the impact of triage-related intervention to enhance patient flow [9,10,11].

Granted these prior reviews focused on patient throughput in various healthcare settings, the majority of these reviews were published before the COVID-19 pandemic and those published in 2020 and 2021 did not assess the impact of COVID-19 on patient throughput. To our knowledge, there is a dearth of systematic reviews assessing the strategies adopted by health care organizations to manage disrupted throughput due to the COVID-19 pandemic. Therefore, the purpose this systematic review is to build on extant patient throughput systematic reviews by focusing on strategies adopted to restore disrupted patient flow due to the COVID-19 pandemic, specifically in the ambulatory care setting. Since healthcare organizations are still dealing with the pandemic, despite the invention of COVID-19 vaccines, a systematic review of published articles regarding outpatient healthcare organizations’ strategies to deal with patient flow during this pandemic would be useful to all health care organizations.

### 1.2. Objectives

The objective of this study is to systematically review the strategies that outpatient healthcare organizations have adopted to mitigate the negative impact of the COVID-19 pandemic on patient throughput and underlying constructs related to patient throughput. Underlying constructs surrounding patient throughput facilitating occurrences/observations (such as continuity of routine care initiatives) and barriers to patient throughput (such as public health physical distancing and isolation measures) are known to exist based upon observations in the healthcare environment. Therefore, identification of best practices and prior organizational experiences for improvement (facilitators and barriers to patient throughput measures) were deemed valuable information as the COVID-19 pandemic continues at a global level.

## 2. Materials and Methods

### 2.1. Eligibility Criteria

Articles included in the review had to meet the initial research database search string criteria, therefore focusing specifically on outpatient/ambulatory care healthcare organizations with patient throughput facilitators and barriers experienced during and in response to the COVID-19 global pandemic. Only quality peer-reviewed, academic journals were utilized in the search initiative. Because only a limited (less than 5) articles identified in the initial search reported on patient outcomes, the research team decided that this was not to be a required criterion for the article to be included in the sample. Articles reporting on patient throughput initiatives during COVID-19 were identified using an aggressive publication date search criteria (1 March 2020 to 1 April 2021) to ensure findings were specifically related to organizations’ responses to the COVID-19 pandemic.

### 2.2. Information Sources

The research team utilized three databases to identify articles in the review: Academic Search Complete, MEDLINE Complete, and Complementary Index. Available via the Elton B. Stephens Company EBSCO library research search website, these three databases were utilized in the study based upon their overall number of results that met the study’s search string and related criteria, while also yielding the fewest number of duplicate articles between databases. All database search efforts were conducted from March 20 through 1 April 2021.

### 2.3. Search

A search string was developed by the research team that involved multiple iterations to generate the highest initial database results in order to be as inclusive as possible, yet also meet the investigation’s intent. Only non-hospital, outpatient (ambulatory care) healthcare organizations were included in the review. The National Library of Medicine’s Medical Subject Headings (MeSH) controlled thesaurus, used to index research articles for PubMed (MEDLINE), was utilized to identify key words for the query search string for the ambulatory care organization terminology.

‘Patient throughput’ and related terms are not included in the MeSH thesaurus. Therefore, the research team conducted various Google searches to identify terms that yielded the most results for this review variable. This initiative was conducted to specifically identify the most common terms and applicable research studies as identified on the Google.com search engine by the review team. Several internet searches were conducted at the individual level by six of the team members and a collaborative webinar was held to identify the most common terms related to ‘patient throughput’ on the web. These terms were then inserted into the search string on the library’s research database.

Multiple database searches were conducted utilizing various Boolean operators to identify the highest initial review sample. String terms were truncated were necessary in order to be as inclusive as possible for specific string vocabulary. The final search string identified by the researchers was: (“ambulatory care” OR “outpatient care” OR “outpatient service*” OR “urgent care” OR “clinic visit*”) AND (“patient flow” OR “patient throughput” OR “waiting time*” OR “wait time*” OR “overcrowding”) AND (“covid-19” OR “Coronavirus” OR “2019-ncov” OR “Sars-Cov-2” OR “cov-19”). This search string was utilized for all three research databases and information regarding the development of each search term is summarized in Table 1. The combination of search terms and their usage in the search string and database search entry fields was a result of multiple search attempts by the research team to identify the search criteria that yielded the highest number of articles identified.

Given that our study was limited to COVID-19-related articles, all published COVID-19 articles were published in 2020–2021, therefore conducting additional search using snowballing was not effective because snowballing led to articles published before COVID-19; these articles did not meet our inclusion criteria.

### 2.4. Initial Study Selection

The Preferred Reporting Items for Systematic Reviews and Meta-Analysis (PRISMA) guided the review process [12]. Six of the seven study researchers participated in the initial database search. Full text was not utilized as an initial search criteria, therefore allowing the maximum number of initial database results. After initial sample article identification, all articles were located in full-text format by the research team and saved to a MS Teams project site. Articles were numbered accordingly and reviewed by all seven members of the research team. A reference management software program was also utilized for citation and PDF storage of the sample.

During the review process, the research team met multiple times via webinar and telephone in order to identify any/all articles from the initial search that met the study criteria. A MS Excel spreadsheet was generated to collectively record underlying constructs identified by the research team (at the individual level), while also providing individual comments regarding each article’s continued inclusion in the review. In all stages of the review process, the review team experienced no disagreements regarding article inclusion decisions or the underlying themes (constructs) identified.

## 3. Results

### 3.1. Study Selection/Exclusion

**Figure 1:**
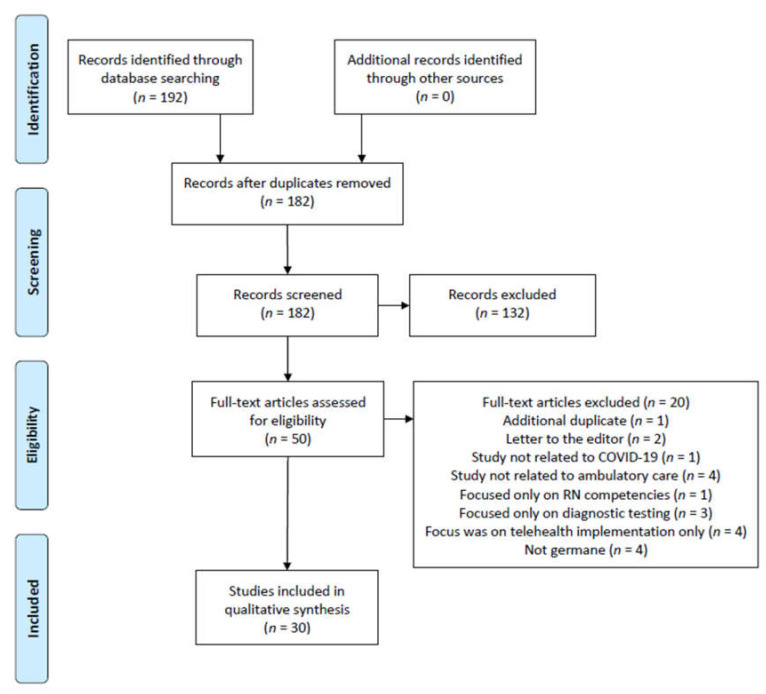
Preferred reporting items for systematic reviews and meta-analysis (PRISMA) figure that demonstrates the study selection process.

The study selection and follow-on exclusion process is demonstrated in Figure 1. The research team’s initial search resulted in identification of 192 articles from all three research databases. A by-database listing of total search results was not recorded (only total records identified through database searching) and therefore is a limitation of the study. Ten duplicate articles were identified in the initial sample and removed. The subsequent filtering process eliminated 130 articles from the initial research database query, leaving 50 articles remaining.

A full-text review of the remaining 50 articles was conducted by the seven-member research team. This was accomplished by five members of the team reviewing 30 articles each. The two other members of the research team reviewed all 50 articles in the findings to assess for eligibility in the review (Table 2). This review effort ensured that each article was read/analyzed by at least four members of the researcher team (minimum).

Upon completion of the full-text review process, an additional 20 articles were removed for the following reasons:

- an additional duplicate article identified (one article)
- letter to the editor (two articles)
- the article was not related to COVID-19 throughput analysis (one article)
- the article was not focused on ambulatory (outpatient) care (four articles)
- the article was a general nursing competency summary (one article)
- the article focused solely on diagnostic testing results (three articles)
- the article focused specifically on implementation of telehealth resources (four articles)
- the article was overall not germane to this review’s research topic (four articles)

Articles removed from the sample for not being focused on ambulatory (outpatient) care focused on overall hospital system(s), hospital emergency department(s), and/or long-term care facilities. Articles eliminated by the research team for the ‘not germane’ reason were completely unrelated to the review’s search criteria yet were somehow identified by the library database search engine. Upon completion of the review, a total of 30 articles were included in the study. Article selection bias was addressed by a series of researcher consensus meetings (via webinar) that focused on each of the 10 articles sets from Table 2. The team experienced no disagreement or difference of opinion in the exclusion of the 20 articles and consensus was established for the remaining articles in the study.

### 3.2. Study Characteristics

The research team’s thorough review of the 30 articles identified underlying constructs (characteristics) associated with patient throughput initiatives (facilitators and barriers) in ambulatory care organizations during the COVID-19 pandemic. The sample included peer-reviewed research articles from several countries/health systems, and the Johns Hopkins Nursing Evidence-Based Practice study design model’s criteria was used to assess study design [13]. Additionally, facilitators and barriers associated with patient throughput initiatives were identified by the research team and are summarized in Table 3.

### 3.3. Risk of Bias

The research team utilized the JHNEBP quality indicator as a tool to assess the quality of each article (strength of evidence) in the sample. This review did not include any level I (experimental study/randomized control trial) articles, which has also been the experience in prior reviews by members of the research team involved in ambulatory/outpatient care organizations [44,45]. This review included nine articles (30%) classified as level II (quasi-experimental) studies and 21 articles (70%) classified as level III (non-experimental, qualitative, or meta-synthesis) studies. A common observation across many sample articles in the review demonstrates outpatient healthcare organizational leaders describing their experiences and convenience samples in attempt to disseminate information regarding best practices and protocols related to patient throughput initiatives. There were no articles identified in the study classified with JHNEBP levels IV or V.

### 3.4. Additional Analysis

**Figure 2:**
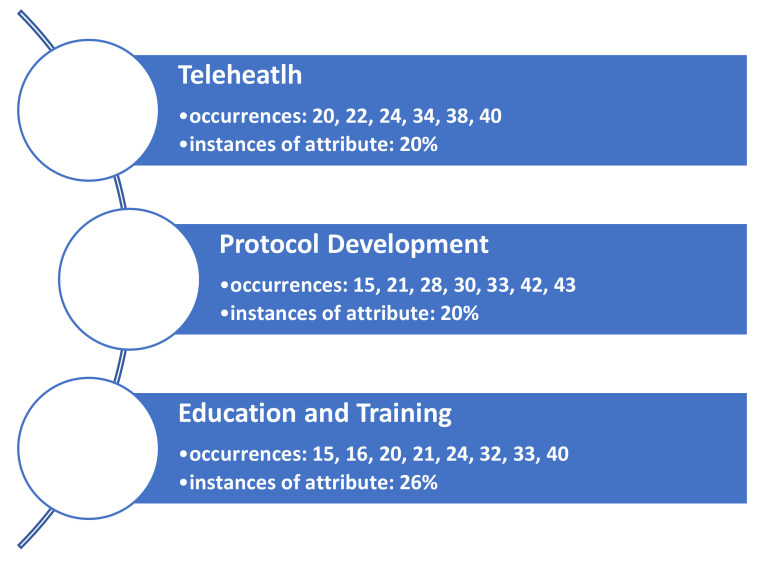
Identified themes (constructs) identified as facilitators leading to an increase in patient throughput in ambulatory care organizations during COVID-19.

Results of the research team’s consensus meetings demonstrate three facilitator themes identified in the literature to support the adoption of telehealth resources for the ambulatory care segment of the industry during the pandemic (Figure 2).

**Figure 3:**
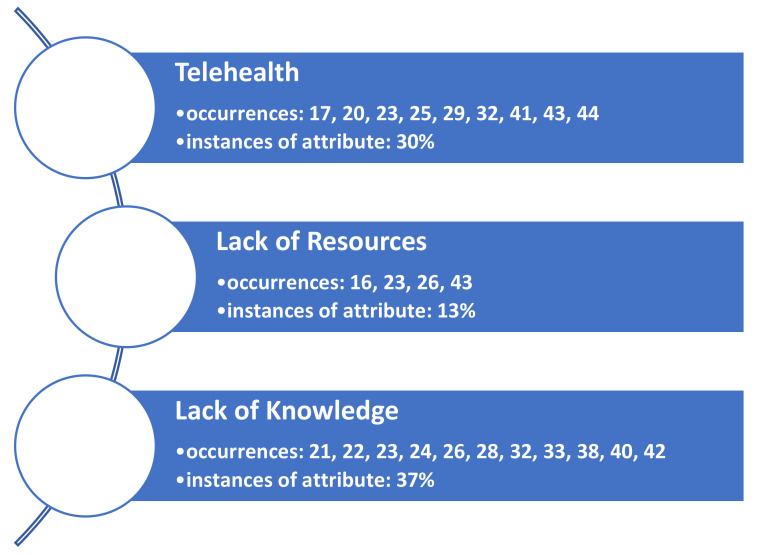
Themes (constructs) identified as barriers leading to a decrease in patient throughput in ambulatory care organizations during COVID-19.

Additionally, three barrier themes were also identified. These are listed in Figure 3. Findings are not mutually exclusive only to a facilitator or a barrier theme, as several articles demonstrated both constructs upon review.

## 4. Discussion

### 4.1. Summary of Evidence

An ongoing initiative to maintain a high level of quality ambulatory/outpatient care continues to challenge the U.S. healthcare system as the COVID-19 pandemic continues. Evidence from this review suggests that enhanced safety and patient distancing protocols [20,27,41], in conjunction with appropriate provider training centered around such public health initiatives [19,23,31]. enhance outpatient providers’ ability to increase patient throughput. Contrary, a lack of both resources [22,25,42] and provider and staff knowledge surrounding updated patient flow protocols [15,22,25] serve as barriers to an enhanced throughput initiative. The review findings also suggest the implementation of additional telehealth initiatives [21,23,33], while also attributing this same identified construct as a barrier to the throughput initiative [19,22,42].

### 4.2. Facilitator to Patient Throughput: Telehealth

The COVID-19 pandemic came with an impact on healthcare resources, services, and budget. The biggest challenge was to evaluate how to minimize the risk of disease exposure and at the same time provide the required medical services. The idea of keeping these patients out of the clinics and hospitals in their healthy state is helping in many ways. Today’s advanced technology is assisting the healthcare industry in the process of managing patients at home. Telemedicine with emails, chat services, text messages, and video-assisted calls between patients and healthcare professionals are helpful in the early diagnosis followed by guidance if there is a need for emergency services.

Implementation of Coronavirus Preparedness and Response Supplemental Appropriations Act allowed pharmacists and other credentialed healthcare providers to offer patient care services via telehealth [39]. This was a collective effort from all the specialized clinics, such as the Uro-Oncology and Neurosurgery Clinics, to develop protocols, staff training, and education and implementation of their knowledge of telehealth regarding patients’ complaints and symptoms [33,37]. A very similar positive effect was seen in a neurosurgery clinic for preoperative and postoperative follow up visits via telehealth during the pandemic [23]. Patients have started to accept telemedicine, which offers safe and effective therapies.

### 4.3. Facilitator to Patient Throughput: Protocol Development

In reviewing the literature, one of the common facilitators identified was the use and development of protocols to help in patient throughput during the COVID 19 pandemic. These protocols ranged from patient check-in to deciding who is appropriate for a needed surgical procedure. The goal of these articles is to make sure patients are cared for safely and resources are used appropriately during the COVID 19 pandemic.

One major reason for protocol development was to ensure that surgical resources are allocated to the correct patient population. Seen in patients in need of orthopedic surgery during the pandemic, protocols were developed to make sure that only necessary emergency surgery were done while others were managed medically until a strain on hospital resources was reduced [27,32]. Another type of protocol that was developed during this time were safety protocols to ensure patients and staff remained safe from the COVID 19 virus. With the implementation of safety protocols for in-person visits, it was found that patients were more likely to stay on treatment regimens with the extra layer of protection these protocols provided [14,27,34,41]. Lastly a protocol theme was identified surrounding how to evaluate staffing levels to ensure that areas in high need of staff were assisted by areas with a lighter workload. Triaging staff was needed to help maintain quality care for patients and for the staff to be able to safely care for an increased patient care load [20,29,41,42]. Overall, there was a positive experience in the development of protocols during the COVID 19 pandemic.

### 4.4. Facilitator to Patient Throughput: Education and Training

Another common facilitator presented in the literature regarding optimizing throughput of patients in the ambulatory care during the pandemic includes education and training components. This applies to both the staff taking care of the patients, along with the patients receiving care. With the ever-changing guidelines presented by the public health authorities and other international agencies, patients need to be educated on the most up-to-date information to keep themselves and their loved one’s safe while being treated in the ambulatory setting [31].

For healthcare staff taking care of patients in the ambulatory setting, education and training pertained to learning new protocols and guidelines, providing education to patients, learning new technology platforms, and conducting research. At times, each day brought new requirements, warranting the creation of new protocols, methods and learning modules for safe patient care [14,15,20,32,39]. For those ambulatory settings conducting care virtually, new technology tools were implemented, requiring training of staff on how to use the new tools, such as Zoom and Microsoft Teams [15,23].

### 4.5. Barrier to Patient Throughput: Telehealth

The U.S. healthcare system was already progressing towards telehealth services intending to provide convenience and comfort for both healthcare providers and patients. The COVID-19 pandemic has helped the healthcare industry in expediting the evolvement of teleservices for medical services and medical education. A transition from physical visits to medical offices and clinics to using telemedicine services was not very smooth in several articles identified in the study. For example, in dermatology patients, about 75% were willing to adapt telehealth for their appointments. However, 25% of patients were still not ready to make the switch. This could be related to patient satisfaction when physicians see patient’s skin ailments and touch their wounds, or it could be the feeling of not getting sufficient attention from healthcare providers because they are not seeing their patients in person [34].

### 4.6. Barrier to Patient Throughput: Lack of Resources

Lack of resources are seen across the healthcare trajectory during the pandemic. There was a concern that patients would possibly be unable to access needed medications to treat medical conditions. Pharmacies lacked resources, patients were not coming in to fill or refill needed medications and there was a fear of medical conditions going untreated [15]. As hospitals were becoming overwhelmed with COVID-19 cases, surgical cases, except for emergency needs, were almost eliminated for a time period. This led to orthopedic ambulatory clinics having to become creative in how they care for their patient population.

Another example of lack of surgical resources was presented by a pediatric urology clinic. A lack of open surgical space and equipment in the hospital led the clinic’s leaders to rethink the way they triaged the patients for surgery. Patients who would experience a life altering effect from not having surgery were offered surgery, but patients who could be treated medically were moved further down on the surgical list [22]. Yet, as the pandemic continues and ambulatory clinics are starting to look towards to a return to somewhat normal operations, there are several concerns surrounding a lack of resources and this particular challenge. Having the proper protocols, guidelines, PPE, and staff resources will have to be considered when looking to return to normal operations [22].

### 4.7. Barrier to Patient Throughput: Lack of Knowledge

As healthcare faced an unprecedented worldwide pandemic, one of the biggest challenges faced was the lack of knowledge of the virus and the effects thereof. Lack of knowledge of protocols and workflow as to how to proceed with orthopedic surgical cases, due to the unprecedented burden brought by the current pandemic, halted surgical cases in healthcare facilities and outpatient clinics. More comprehensive guidelines for the orthopedic surgeon in the era of COVID-19 were needed to maintain a safe and effective practice to resume surgical cases [27].

As the challenge to care for patients in the outpatient setting continued, more data was gathered and safety guidelines and protocols were created, but healthcare providers would find out when implemented that they may not meet the needs of patients. For instance, one radiation oncology clinic surveyed their lung cancer population to see what effect the pandemic was having on their treatment schedules [25]. This provided a method to obtain patients’ point of view on how the pandemic has changed their perceived access to treatment. It was identified that there was an increase of wait times for treatments and an increase of fear of contracting the virus, which held them from seeking such treatment [25]. One of the greatest unknowns of the pandemic for the ambulatory space was the simple fact of seeing patients safely and being cognizant of the resources available for treatment [31,37,39]. This lack of knowledge of telemedicine prior to COVID-19 led to an overwhelmed healthcare industry unable to keep up with demand for outpatient, elective services, pharmacy services, neurosurgery, pediatric otolaryngology, cancer patients, and pain treatment patients [21,23,25,33]. Although much of this was remedied using telemedicine and virtual clinics, there was a large learning curve, and it was unknown at the time if these clinics would offer the same quality as an in-person visit.

The findings from this systematic review are not directly comparable with prior systematic reviews on patient throughput given our special focus on COVID-19, which has dramatically disrupted patient throughput. Since COVID-19 is the major cause of disruption, throughput strategies such as the use of telemedicine, protocol development, education and training were especially implemented to address that disruption. Therefore, these strategies and related characteristics were not found in prior systematic reviews focused specifically on patient throughput. Pre-COVID-19 strategies to improve throughput that were found in systematic reviews consisted of the use of medical scribes [4,5], the use of lean management and process improvement [2,3,6], the use of triage liaison [10], and the role of nurses [11]. Additional studies focused on the establishment of access centers, call centers, patient placement centers, ED clinical laboratory, ED self-registration kiosks, ED result waiting area, physician-assisted triage, patient flow automation, and rapid admissions units [2,7], the use of hospital staff to undertake responsibility for ED admissions [2], and admissions screening for MRSA [2]. Lastly, the expansion of nursing roles such as clinical initiative nurses and nurse practitioners and fast tracking the care of clinically stable patients are identified as constructs prior to the COVID-19 pandemic that also support initiatives to accommodate patient throughput [7].

## 5. Conclusions

Across the globe, patient care processes and related public health disease precautions have changed to adapt to the ongoing COVID-19 pandemic. This systematic review identified facilitators and barriers related to patient throughput initiatives in ambulatory care (outpatient) organizations. Identified constructs from this study support ongoing patient care initiatives and offer insight into additional, future efforts towards the continuity of outpatient care during the pandemic.

Telemedicine, identified as both a facilitator and barrier to patient throughput in the outpatient setting, demonstrates how increased access to medical providers for consultations and related care efforts can be improved, while high-touch, in-person care processes are lacking. Further facilitating of outpatient organization throughput concerned planning and strategic implementation of protocol development processes/procedures and related education and training initiatives as adaptive initiatives. Patient throughput barriers (in addition to telemedicine) regarding organizational needs (PPE, clinic space for physical distancing, and medical staff) and overall lack of knowledge surrounding patient care processes related to throughput initiatives were also identified. Ambulatory care providers can benefit from these patient throughput facilitators and barriers as the global pandemic continues. Future research surrounding these identified constructs include specific outpatient industry segment care lines (specialty care) and associated patient outcomes within and between care processes.

As with most systematic reviews, this study has some limitations. First, given that we reviewed published studies on COVID-19 patients throughput, and since most COVID-19 studies were published in 2020–2021, conducting additional article search using a snowballing method was not effective since snowballing leads to articles published before 2020 before the COVID-19 pandemic. Second, we did not record the number of original articles by database since by-database listing of original findings is an optional step in the PRISMA guideline.

## Figures and Tables

**Table 1:** Development and Use of Search Terms.

Search Variable	Development Method	Database Usage
Ambulatory Care	MeSH (exploded)	Subject Terms (SU)
Patient Throughput	Google search	All Text (TX)
COVID-19	EBSCO research database popular terms list	Subject Terms (SU)

**Table 2:** Reviewer assignment of the initial database search findings (full article review).

Article Assignment	Reviewer 1	Reviewer 2	Reviewer 3	Reviewer 4	Reviewer 5	Reviewer 6	Reviewer 7
Articles 1–10	X	X	X	X		X	X
Articles 11–20	X	X	X		X	X	X
Articles 21–30	X	X	X			X	X
Articles 31–40				X	X	X	X
Articles 41–50				X	X	X	X

**Table 3:** Summary of findings (*n* = 30).

Author(s)	Participant(s)	JHNEBP Study Design *	Facilitators Leading to an Increase in Patient Throughput in Ambulatory Care Organizations during COVID-19	Barriers Leading to a Decrease in Patient Throughput in Ambulatory Care Organizations during COVID-19
Akuamoa-Boateng et al. [14]	German University Hospital radiation oncology clinic	3	Changing workflow designs and patient selection led to reduced first-contact appointments and significantly increased downstream appointment compliance. Observation of pre-Covid clinic flow including barriers and compared them to during COVID clinic flow with increased precautions and looked for areas to optimize. Having an “active flow management” for each patient helped patients stay on treatment and helped physicians not have to delay future patients from treatment planning.	Non-treatment-related routine follow-up appointments were deferred in mutual agreement with patients and rescheduled within 2 to 4 months in close consultation with the primary oncology care giver. Alternative active patient flow management procedures were prepared by installing a hermetically sealed infrastructure and exclusively assigned personnel governed by security concepts.
Anderson et al. [15]	Ambulatory care pharmacy preceptors in the U.S.	3	Physician/pharmacy care team would ultimately communicate all decisions back to the patient, being mindful to limit the number of people in a single care room. Physical examinations or monitoring tests (vital signs or other point-of-care tests) were conducted by the pharmacist or year 2 ambulatory care residents, whereas student clinicians had less-acute conditions to treat to help with process flow.	Learner removed from onsite, reducing their ability to treat in-person. Findings concluded that while there are multiple methods of changing the delivery of care, ultimately the methods need to evolve more to continue addressing the challenges COVID-19 has given the healthcare system for patient care. This is even more true with preceptors and students.
Aquilanti et al. [16]	Dental patients in Italy	3	Trust in dentists regarding sanitization procedures and perception of the impact of the risk of contagion on dental care impact the patient compliance/no-show rates.	Fear and anxiety generated by the spread of the virus will impact more than the lowered familiar income with regards to access to dental care.
Atchley et al. [17]	U.S. nurse practitioner outpatient clinics	3	Sick patients were triaged via telephone. Social distancing was maintained by either seeing patients at well-spaced out intervals or in some cases assessing and treating patients in their personal vehicles in the clinic parking lot. Ensuring open communication channels among the various staff to discuss needed changes and feedback from patients can both support the creation of a culture of change and safety within practices and help reduce wait times. Appropriate staff use, good scheduling practices, and maximization of time spent by patients in the clinic, combined with the integration of telehealth care, can improve clinic flow and reduce wait times. By reducing wait times, providers can reduce costs while improving patient outcomes and perceptions of care.	Many patients were sheltering in place at home during this time to decrease the possibility of exposure to the COVID-19 virus. State and local governments invoked recommendations restricting nonessential services, including well and routine healthcare visits.
Baughman et al. [18]	Boston post-acute care facilities and surrounding healthcare organizations	3	Local government and health care leaders collaborated to rapidly establish a 1000-bed field hospital for long-term care patients. COVID positive patients were transferred to a local health care organization for the homeless with 500 respite beds for required isolation. Centralized, large nonprofit multicenter health care system provided financial, operational, and human resources to develop and manage beds for patients with COVID-19 requiring transitional or respite care from hospitals and outpatient settings. Partnership with local government, military, and major health care organizations was essential for logistical and medical resource support.	Admissions were limited by patient perception that the field hospital was more of a shelter rather than a post-acute care hospital and other concerns around general comfort, privacy, and the no-visitor policy. Not all beds were utilized, as only 394 patients were admitted to the field hospital.
Beattie et al. [19]	Inner Hebrides of Scotland outpatient clinics	3	Enabled video consultations with specialists to take place in the patient’s home. This study reaffirms the view that patients and the public indeed hold unique perspectives about how health services can be designed to fit their communities. The project successfully codesigned the use of Near Me at Home video consulting, through quality improvement methodologies to address a key issue for the community of Skye.	n/a
Casiraghi et al. [20]	Spedali Civili Italian hospital trauma department patients	3	Redistribution of human and technological resources to pneumology, infectious disease, and intensive care increased productivity of the trauma unit. Three “hub” hospitals for major trauma were identified in the region for these specific types of patient. All trauma activities that could not be postponed were concentrated in this trauma hub. Adaptive staging based on patient COVID status at the time of treatment was created to help improve workflow processes, while protecting patients and providers.	In order to leave the red zone, all healthcare professionals stepped over a puff embedded with chloro-derivate solution. Other stage-related precautionary measures did slow workflow processes. Creation of a COVID-positive and COVID-negative surgical floor resulted in an imbalance patients on either floor at any one time, resulting in redistribution of resources to accommodate workflow needs.
Darr et al. [21]	NHS tertiary pediatric referral center	2	Use of virtual outpatient clinic encounters for pediatric otolaryngology assessments resulted in 99% initial diagnosis accuracy. Findings demonstrate a positive response to the addition of the telehealth with less cancellations, increased referral back to primary care and a decrease in planned surgical procedures.	The use of aerosol generating procedures (AGPs), particularly flexible nasendoscopy (FNE) was minimized, with recommendations for use only in extenuating circumstances. Postponement of most elective outpatient and inpatient services occurred per local/regional government policy recommendations. A need for a detailed examination was still identified after a virtual visit. Use of instrumentation and further investigations limited the use of virtual visits, necessitating follow-on face-to-face appointments based on the clinical priority level.
Das [22]	Community-based ambulatory endoscopy center in the U.S.	3	n/a	Post–COVID-19 recommended workflow changes significantly impacted the operational and productivity metrics and, in turn, adversely affected the financial metrics. With the addition of COVID 19 procedures, increased time and costs for the patient and center occurred. There was a significant increase in total processing times, waiting times with a consequent decrease in productivity, and financial metrics precisely because of a bottleneck at the time of pre-procedure COVID-19 screening and testing while practicing social distancing. Incorporation of recommended post–COVID-19 related workflow modifications adversely impacted the efficiency and utilization of an AEC across a wide array of performance indicators.
De Biase et al. [23]	Tertiary institution neurology clinic (U.S)	2	Telemedicine capability is more widely accessible with lower technological barriers to adoption at this time.	Neurosurgical practices were negatively affected by the government mandates to cease elective surgeries combined with national stay-at-home orders, resulting in a considerable drop in outpatient visits.
dos Santos et al. [24]	Public university service mastology outpatient clinic in Ceará	3	Identification of scheduled patients, reading of clinical developments in electronic medical records, individual assessment to define whether or not appointment would remain, telephone contact to inform about unscheduling helped improve operations.	The number of outpatient users is high, which normally causes crowds in the corridors. Increased COVID-19 cases brought the need to restructure healthcare services. Lack of time to follow up service was a limitation of this study.
Fu et al. [25]	Lung Cancer patients at a health system clinic in the People’s Republic of China	2	n/a	An increase in wait times and a decreased access to care was due to an increase in need from COVID 19 patients. There was also a decrease in care due lung patients’ fear of contracting COVID19.
George et al. [26]	Singapore community health pain management clinics	3	Close partnership between pain specialists and community nurses to collaboratively adopt a systematic and comprehensive approach to assessment, treatment compliance and outcome monitoring. Patients with impaired mobility, poor social support and multiple comorbidities, especially older adults, were considered for referral to community teams. Teleconsultation now recognized as a feasible solution, allowing assessment and social interaction and shortening the waiting times to consultations while fulfilling the requirements of social distancing. Community volunteers assisted patients to be digitally connected with their health and social care providers by improve accessibility to mobile devices and information technology literacy. Overall, the integration of community healthcare teams into the holistic, long-term management plans for vulnerable patients with chronic pain increased patient throughput and overall care.	Some barriers with patients who were not able to receive treatment for their comorbidities other than pain. Telemedicine has legal and safety limitations in monitoring opioid consumption. Community services such as home personal care and center-based care services were scaled down. Face-to-face visits were limited to 30 min. Older people and the less ‘tech savvy’ among the pain clinic’s patients were not open to the concept of video-consults initially, preferring telephone interviews and face-to-face consultations.
Gharaibeh et al. [27]	International orthopedic clinics	3	Orthopedic surgery workflow (zones) created for patients having surgery to safely implement COVID protocols. Outpatient clinics established a provider testing system to ensure they do not spread COVID in the outpatient setting. Part of the clinical assessment such as history taking, may be completed using a digital interface to limit the interaction between the patient and the medical staff. An ‘off-duty’ team can use Telehealth to manage remote follow ups to reduce the burden on the active team or health care system.	High-risk outpatients follow a scheduling protocol that automatically establishes a 14-day waiting period. Designated, separate operating suites created for COVID and non-COVID patients (pre-established). Intubation/extubation is to be performed in a separate area from the operating room. Surgical/OR patients are to have a reduced surgical team in order to decrease the movement of individuals and prevent spread of the disease, increasing individual workload in the delivery of care.
Hockaday et al. [28]	Federal Medical Station for COVID patients in Dallas, TX, USA	3	Methods of PPE conservation, while attempting to maximize staff safety by using defined protocols allowed for continue patient care.	When removal of masks in the patient care areas is required, the affected person should immediately mobilize toward the doffing zone exit, maintaining a minimum distance of 6 ft from all staff and patients.
Janig et al. [29]	Military medical treatment facilities	3	Established military medicine protocol involves ongoing assessment of available resources and transfer the patient to the highest level of care available if the patient’s status permits. MEDEVAC (helicopter) evacuation protocol exists to also remove the COVID positive patient from theater immediately. Initial or re-evaluation of the patient in theater requires an assessment of COVID-related symptoms to be included in the triage and treatment decision algorithm established in the author’s protocol.	Availability (or lack of) ICU care for soldiers in theater significantly impacts access to care, even during the COVID pandemic.
Küçük et al. [30]	Health Ministry of Turkey EHR/EMR data from multiple healthcare organizations	2	Use of appointment systems has become more important in order to minimize the risks of spreading COVID-19. Appointment scheduling systems demonstrated positive impact on waiting times.	Scheduling problems, no free time slots available for physicians, and physician or hospital-related problems slowed patient flow. The scheduling system had many barriers, such as health policy implications in Turkey, preventing full implementation.
Kyari & Watts [31]	U.S. outpatient eye clinics	3	Scheduling adaptations to have specific types of eye patients arrive for care at established time periods allows for increased throughput. Encourage patients to not bring family members/others with them to their appointments. Wayfinding/people moving systems (one-way paths) throughout the clinic for patient-flow and physical distancing enable better workflow. Use of clear and interpretable images (chair markings, etc.) assist in physical distancing communications and related messaging in the eye clinic.	Fragile health systems will return to the new normal in a less unified/organized manner. Where there have been no established social protection schemes, the response will be slower, and even more difficult for eye clinics without established protocols. An ongoing review of national and local updates (policy) to be implemented will alter clinic productivity.
Lou et al. [32]	Orthopedic surgery institution in Shanghai, China	3	Professional organizations provided recommendations/guidelines on how best to manage selective operation patients during post-epidemic period. Strict enforcement resulted in better workflow and less spread of COVID across providers. A developed workflow they returned to pre-pandemic levels of orthopedic cases and they were able to handle them safely. Spread of COVID reduced by a stepwise strategy with a sound screening system, a combination of various diagnostic methods and appropriate personal protection to facilitate workflow.	Precautions to manage elective surgeries might be considered unnecessarily costly, overly rigid and time-consuming for a region that has cleared its local infected cases for months. Possible false-negative results for RT-PCR tests resulted in a proportion of asymptomatic or pre-symptomatic COVID-19 patients testing negative; these patients could be potential drivers of viral spreading. Further chest x-ray/diagnostics required that slowed workflow. Orthopedic procedures prone to generate aerosol, raising the potential risk of viral transmission in operating theater.
Lynch et al. [33]	Representatives from adult pain clinics in Canada	2	They found that the added benefit of telehealth was beneficial to patients with chronic pain issues during the pandemic in helping keep care on track. Most telehealth care offered was for follow-up and maintenance of ongoing care for routine patients only.	Survey feedback demonstrated that regardless of tele-health (phone, webinar) options offered by pain management clinics, patient throughput still slowed as patients were reported to have to wait longer than normal for their care. Many patients without access to other diagnostic or therapeutic interventional procedures (urgent pain care offered by providers only during the pandemic). Alternative/complementary therapies in conjunction with regular pain management care was halted.
Mason et al. [34]	Radiotherapy patients at the The Christie at Oldham satellite center in the UK	3	Designated areas for staff for putting on and removal of PPE. Development of a designated COVID-19 proforma to support telephone triage of patients telephoning with possible symptoms. Patients who themselves are asymptomatic but need to self-isolate due to contact with someone who is symptomatic or confirmed COVID-19, are treated at the end of the day. Patients’ relatives or carers are discouraged from attending with the patient for their radiotherapy appointment. Review of patient scheduling so the department treats at the most risk patient groups in the morning on both linear accelerators.	n/a
Mukerji et al. [35]	Otolaryngology clinic at a U.S. community pediatric hospital	3	Rotation schedule for providers and ancillary staff. Guidelines for in-clinic visits and alteration to surgical block and surgical case cadence. Ongoing algorithm workflow revisions were made at each phase of the pandemic related to in-clinic visits, telemedicine visits, and surgical cases for best outcomes/volume. Team A was designated as the “Urgent” team and Team B was the “Home” (telehealth) Team. Social distancing and prevent cross contamination we designated one area as the “urgent” clinical area and assigned one exam room and one procedure room for the urgent provider to see patients and perform clinical procedures. The “clean area” was used to perform telehealth visits with appropriate social distancing.	Otolaryngologists and pediatric otolaryngologists are amongst sub-specialties with an increased risk of exposure to COVID-19. Only one caretaker was permitted to enter the hospital with the patient. A “slow ramp” up phase was required, and limiting clinical templates were opened to provide in-clinic patient care. After a physician performed an aerosol generating procedure, the provider placed the laryngoscope in a biohazard bag and the procedure room was then closed for 1 h. Social distancing was optimized by increasing the turn-over time between procedures.
Raidla et al. [36]	Hospital system in Sweden	2	Creation of a primary care-like facility in close proximity to the hospitals may relieve overcrowding of the hospital’s ED, especially during COVID-19. Having patients triaged appropriately to the Urgent Care Center helped save patients time and money and helped save the health system in time and they saw a reduction of resource overutilization. Provider familiarity with the facility in which they work and the devices they need to use essential.	The ED staff may be more focused on recent symptoms and a rapidly emerging illness. The urgent care clinic staff be focused on long-term medical history.
Rodler et al. [37]	Patients currently being treated for genitourinary cancers at a single German hospital	2	Findings showed that there was low risk of this patient population to contract COVID-19 if all protocol was followed. However, it did suggest the option of telemedicine for care to maintain patient care. Significant, early precautionary steps (physical distancing and other patient-acuity level protocols) enabled a low infection spread/rate and kept the clinic open. Multidisciplinary tumor boards for treatment decisions were transformed to teleconferences or video conferences. Virtual management and reductions in frequency of visits are feasible and will likely impact the future treatment approach of patients with genitourinary cancers after the crisis.	Strict quarantine of specific patient acuity types was part of the protocol, impeding care processes at the clinic level (while using telehealth resources). All clinical trials were paused.
Sacchelli et al. [38]	Psoriasis patients in ambulatory care clinics in Italy	2	Psoriasis providers recommend making patients more confident in their services and safety provisions, encouraging them to refer/attend appointments In the case of clinical/therapeutic doubts, achieving a better compliance to treatment is recommended by working to ensure patient safety and control of misinformation via patient-provider communications.	Misinformation (termed ‘info-demic’) spread rapidly during the pandemic and changed the clinical course of patients with severe psoriasis. Many patients stopped their psoriasis treatment during lock down as a result. Word-of-mouth (often family member) recommendations to stop psoriasis treatment during the pandemic resulted in frequent appointment cancellations.
Segal et al. [39]	Washington state pharmacy service for multiple ambulatory care clinics	3	An expedited telehealth program was able to be fast tracked due to the relaxation of CMS guidelines of telehealth regulations. Telehealth visits are preferred over phone visits to ensure patient understanding and to help establish the pharmacist and/or care team establish and build rapport with the patient, eliminating unnecessary in-person follow-up appointments. Expansion of telehealth eligibly pharmacy services patients (not just for rural health and other patient categories).	The telehealth visits resembled a scheduled in-person appointment format. For telehealth to be successful, both parties must stick to their scheduled appointment time. For pharmacists working off-site, wireless Internet seems less stable and slowed process workflow.
Tam et al. [40]	Cardiac health system in Ontario that has outpatient clinics	3	A triage system for patients for was enacted to determine appropriate cardiac care during the pandemic and how to properly gauge the use of resources to slowly reopen to a larger capacity. A proper workflow is needed to balance the need for cardiac care during the pandemic and current COVID-19 patient loads.	COVID-19 patients and cardiovascular patients compete for the same resources and this affects workflow negatively for both groups.
Thorakkattil et al. [41]	Johns Hopkins Aramco Health Care (JHAH) ambulatory care pharmacy services in Saudi Arabia	2	Staff schedule rotations and allocations were applied to reduce the number of available staff per pharmacy unit to enable appropriate physical distancing and to cater for the expanded staffing needs of the call center and the additional temporary pickup locations. Through the use of home delivery, off site medication pick up and online portal medication requests, the pharmacy was able to maintain quality in the care offered and honored the infection protocols. Encouraging patients to use the remote pickup locations of JHAH pharmacies helped.	Considerations had to be made for the consequences of governmental decisions (e.g., curfew, areas in lockdown, and stoppages of transportation services.
Wang et al. [42]	Outpatient fever clinics located at the Union Medical College Hospital (China)	3	An evaluation of the effect of upgrading the fever clinic system assisted with rates of nosocomial COVID-19 infection and (ED) emergency department patient attendance at Peking Union Medical College Hospital. The workload of the FC increased significantly after the COVID-19 outbreak and new protocols regarding the use of the fever clinic likely helped prevent the spread of COVID-19 within the hospital and reduced further burden on the ED.	n/a
Waya et al. [43]	African healthcare organizations	3	Asymptomatic, mild and moderate cases without comorbidities or risk factors are isolated and managed at home, with symptomatic management for mild and moderate cases and close monitoring for any clinical deterioration. African governments and scientists should strengthen national capacities for the generation of local evidence which could guide the development of home-grown case management strategies, protocols and equipment for the management of COVID-19 cases on the continent. Home-grown, community-specific protocols assist with preventing COVID spread to healthcare providers while also avoiding social stigmas.	Facility-based isolation of COVID-19 cases extremely limited, given the health infrastructure and health workforce issues in Africa, including the risk of nosocomial transmission. Poor housing, overcrowding, inadequate access to water and sanitation, and stigma related to infectious disease that is prevalent in many African societies was not an option an further slowed organizational processes.

* Johns Hopkins Nursing Evidence-Based Practice (JHNEBP) levels of strength of evidence: Level 1, experimental study/randomized control trial (RCT); Level 2, quasi-experimental study; Level 3, non-experimental, qualitative, or meta-synthesis study; Level 4, opinion of nationally recognized experts based on research evidence/consensus panels; Level 5, opinions of industry experts not based on research evidence.

## Data Availability

Not applicable.
